# Medico-Chirurgical Transactions

**Published:** 1816-06

**Authors:** 


### PART II.

### COMPREHENSIVE ANALYTICAL REVIEW OF MEDICAL LITERATURE.

"Tros, tyriusve, *nobis* nullo discrimine agetur."

### *Medico-Chirurgical Transactions, Vol. vi. pp.* 676. Six Plates. London, 1815.

The Members and Contributors of this Society are rapidly increasing, and two volumes per annum are in future to be published. The expence, however, of the volumes will operate as a very considerable check on their circulation through the minute ramifications of the profession, and their utility will be thereby abridged. We shall endeavour, in some measure, to remedy this evil by increased exertion in our analytical labours; and we hope to let no useful fact or ingenious suggestion contained in these Transactions remain unnoticed, or beyond the reach of every member of the faculty.

The papers are very unequal, both in value and bulk in this volume; and we shall exercise our discrimination in apportioning our allotted spaces accordingly.

I. The first Article is by Dr. Calvert on the Plague at Malta in 1813; it occupies sixty three pages, and its whole substance may be very easily compressed into little more than as many lines. A vessel called the "San Nicholo" from Alexandria, arrived at Malta on the 29th of March, having lost two men on the voyage by the plague. She was put in quarantine, and the crew confined in the lazarett, where the master and his servant died on the 2d of April of the plague. Notwithstanding every precaution, the disease appeared soon afterwards on shore, where it continued to radiate, and immolate its victims during nearly the remainder of the year. Forty-four pages are taken up in tracing the plague from house to house, from street to street, from family to family, or in shewing how the disease broke out in various and distant quarters, where no personal communication could be proved, or hardly suspected. At length comes the whole drift of this *lengthy* paper.

"I do not mean to deny that contact generates the disease; on the contrary I am persuaded, that this or vicinity, particularly if long continued, is the most certain mode of communicating it, as the history of the progress of the plague at Malta sufficiently illustrates; but I am inclined to deny that this is essential to the propagation of the contagion. It appears to me, that this contagion or principle of plague, is diffusible in the atmosphere to a distance greater or less from an infected body, according to the climate and season of the year, and possibly, to other peculiar states of the atmosphere, with which we are unacquainted: that in the spring or summer season, a single infected is sufficient to contaminate the air of a whole city; and that those who happen to be then exposed to febrile causes, or otherwise predisposed, are the first to become its victims. That these newly infected persons generate a fresh supply of poison, increasing its strength and influence, till at length it becomes so powerful, that nothing but the winter season will entirely put a stop to it. Nor is this wide diffusibility of the pestilential poison from the body of one man more wonderful, than that of a grain of musk, that will *sensibly* affect the air in a room for twenty years together, without suffering any diminution of its volume. In this way, and in this way alone are we able to explain the first introduction of the plague into Malta, in the year 1813."

The practical deduction from Dr. Calvert's theory is, that the lazarets should be erected at a great distance from human habitations, in order that the contagion of plague may not be able to permeate the atmosphere and propagate itself among the inhabitants.

We are much afraid that this theory of Dr. Calverts will not, like the grain of musk, be found to *sensibly* affect the medical and political horizon for twenty years together, "without suffering any diminution of its volume." It is a theory calculated to inspire terror, and destroy confidence; nor do we conceive the data, on which he builds his hypothesis, at all adequate to the support of the superstructure. Nothing is saidof the nature or treatment of the disease itself; and upon the whole, we consider this *Article* of sixty-three pages in a guinea volume, as one of the *dearest* we have purchased for many years.

ART. 2.—*Case of Locked Jaw cured by Oil of Turpentine in Glyster.* By EDWARD PHILLIPS, M. D.

Miss S. a very delicate and sensitive young lady, ætat. 20, and subject for some months previously to spasmodic affections, was seized on the 22d of August with strong and general convulsions. "The jaw was firmly locked, the whole of the left side paralytic" with frequent unavailing attempts to vomit after each abatement of spasm. She could not discern one friend from another; yet there was no affection of the pupil, nor of the vascular system. This state had continued 24 hours before she was visited. The warm bath; an enema, with salts and senna afterwards. 23d. Relief was experienced from the warm bath, with mitigation of sickness; no stool from the enema. She wrote on a slate that there was acute pain in the right side, near the region of the liver, with soreness on pressure. Pulse undisturbed; jaw locked; leeches to the side. Dfts. of infus. rosæ et magnes. sulphat.; balneum calidum vespere. 24th. No material alteration, except abatement of pain in the side. As the evacuations were scanty, scammon. ℈j; sub. hyd. gr. viij, statim sumend. Vespere, The convulsive paroxysm more severe than ever. Venæsectio ad ℥x, with some relief. Warm plaster to the stomach, and anodyne draught at bed-time. 25th. Slept great part of the night Spasms relaxed; sickness and trismus still continued. Complained of acute head-ache; leeches applied to temples; repeated the purgative powder. No feeling or motion in the left side. No urine the last 24 hours. 26th. Bowels had been freely opened; spasms less frequent or severe; no sickness of stomach. At seven in the evening, a more violent recurrence of all the symptoms than ever; to which were added syncope; weak, rapid, and intermitting pulse, with incessant sickness. In this distressing situation "I immediately desired, says Dr. P. that a glyster might be thrown up with some force, from a syringe, composed of half an ounce of the oil of turpentine, rubbed down with the yolk of an egg, in eight ounces of infusion of senna. In about five minutes after the glyster was given, we were in a hurried manner called to Miss S. whom, to our great joy and surprize, we found sitting up in the bed smiling; the jaw was completely unlocked, and she with great complacency, thanked us for the great, and almost instantaneous relief we had afforded her. She was able to move the left arm and leg, and there was a total subsidence of the disease, and its attending bad symptoms." The next day she passed a copious stool, in which was a small worm. The young lady described her sensations during the exhibition of the enema thus:

"Immediately after it was given, she felt a glow of heat, accompanied with a prickling sensation, first in the calves of both legs, pursuing the course of the spine up to the neck, and afterwards to the head and face; the room then appeared to her to be full of smoak, and the jaw instantly fell." 71.

We consider this wonderful cure of trismus, by half an ounce of oil of turpentine in a glyster, as barely a sufficient inducement to try the remedy again; for we who have seen a few of these distressing complaints, and know their obstinacy and fatality, put no confidence whatever in the remedy in question. The case of this *sensitive* young lady was a peculiar one altogether, and in our opinion, one of intestinal irritation acting on a very susceptible nervous system. Much do we fear, that the same remedy will rarely be found again to produce the same effects in trismus!

Art. 3.—*Case of an extraordinary Enlargement of the Scrotum, with an Operation successfully performed for its Removal.* By John Maddox Titley, M. D. of St. Christopher's.

Dr. Titley represents elephantiasis as having lately spread with unexampled rapidity through the whole of the West Indies, especially among the blacks; and affecting not only the lower extremities, but the arms, penis, scrotum, and even the viscera.

Monsenatt, a negro, received the kick of a mule on the right testicle, when a boy, after which he was subject to the rose in the legs. Some years afterwards, the scrotum swelled, and the disease went on progressively. The following is the description of the complaint in October last.

"On removing his petticoat, there was exposed to view, a tumor of rather an oval form, seemingly suspended from, and greatly stretching the abdominal integuments and spermatic cords, reaching backwards to the verge of the anus, and descending to within an inch or two of the ground. It measured longitudinally, from the symphysis pubis to its base twenty nine inches, circularly forty-three. The spermatic cords could be distinctly felt, somewhat enlarged, the penis was completely enveloped; the urine was discharged in a full stream, and without difficulty, at an orifice situated nine inches below the pubis; on stretching this laterally, the extremity of the penis could be seen at about the distance of three inches; this canal was formed by an elongation and distention of the prepuce. The surface of the tumor was equal, smooth, with superficial veins; the superior part thinly interspersed with hair; the inferior scaly at times. The integuments felt extremely thickened, but were not of equal firmness, and retained for a time the impression of the finger. His appetite was good, his bowels regular, and his general health unimpaired. He informed me, that when on his back in bed, and under the impression of lascivious ideas, he was subject to erections of the penis, at which times this member would project an inch or two at the orifice above mentioned; but that they were never terminated or attended by seminal emissions." P. 76.

"*Operation.*—The only difficulty that seemed to occur was, with regard to the possibility of saving the penis; with a view to effect this, an incision two or three inches in length was made, commencing from a little below the symphysis pubis; the penis was by this means exposed, and the extended præputium being divided, a flexible catheter was introduced into the bladder; all our efforts to accomplish this having previously failed, owing to the retrocession of the penis. The spermatic vessels of each side were next laid bare and secured by temporary ligatures passed round the cords. The incision was continued backwards to the verge of the anus, and the dissection carried upwards towards the penis. The tumor being removed, the spermatic arteries were secured separately, and the upper ligature slackened, but allowed to remain in case of need. The integuments were brought together by a few stitches and strips of adhesive plaster, and were sufficient to surround the root of the penis; so that this member was the only part that remained uncovered. The hæmorrhage during the operation was very inconsiderable, (except from a branch of the left pudic) the precaution being afterward used of passing a ligature round the large arteries, previous to dividing them. My patient recovered without experiencing the most trifling unpleasant symptom. The wounds in the groins and in perinæo, were firmly united at the end of three weeks, but the penis was not completely cicatrized before the beginning of April." P. 78.

On examining the tumor after its removal, the testicles were found to occupy their natural position; the left one was about the size of a hen's egg; the tunica vaginalis on the right side contained three pints of water, and the testicle was considerably diminished. The right side of the scrotum being opened, the integuments at the upper part were about two inches; nearer the base, they increased to four inches and a half in thickness. A fluid oozed from its substance, and the cavity was filled with a gelatinous matter.

Art. 4.—*On the Use of Nicotiana in Retention of Urine.* By Henry Earle, Esq. Surgeon to the Foundling Hospital.

The causes of retention of Urine are various, and consequently the remedy here brought forward is not meant to apply to any but a particular class of these complaints. In organic stricture of the urethra, the passage of the urine is sometimes totally stopt by a *spasmodic* affection of the muscles of the urethra, or inflammation of that membrane. The same takes place in persons labouring under stricture, by retaining their urine beyond the usual period of expulsion. In irritable states of the urethra, it is often imprudent to introduce an instrument when there is suppression of urine; and in such cases, purgatives; general and local bleeding; warm baths; and tinct. ferri mur. are resorted to. Occasionally, however, these fail; and here Mr. Earle presents us with another powerful auxiliary exhibited as an enema, in the form either of smoke or infusion.

Three cases are related where suppression of urine resulting from organic stricture combined with spasm was relieved, after many other means failed, by the infus. nicotianæ (ʒj to eight ounces of water) thrown up the rectum. It generally occasioned faintness, perspiration, and sometimess, sickness, after which the urine flowed.

Art. 5.—*Case of Obstruction in the large Intestines, occasioned by a biliary Calculus of extraordinary Dimensions.* By H. L. Thomas, Esq. &c. &c.

Mr. Thomas was called to an old woman labouring under the usual symptoms of obstructed bowels, and who had an irreducible umbilical hernia as large as a melon. There was considerable tension of the abdomen, with pain on pressure, particularly above the left ileum; nausea and vomiting of bilious matters; pulse small and frequent; countenance indicative of distress; surface of the body bedew d with cold clammy perspiration; no fæcal evacuation for some days. Mr. Thomas proposed the operation for strangulated hernia, but it was not acceded to; ten grains of calomel were given; an opiate glyster ordered, and a hot fomentation of brandy and vinegar to the abdomen. In the night, the vomiting ceased; the skin became warm and perspirable; the pulse quieter; and in every other respect she was more composed. For two days the symptoms seemed to moderate; but no stool was passed. On the third day, her torments seemed to increase with redoubled violence, when an evacuation suddenly took place, preceded by an extraordinary noise, as though some solid body had been forcibly projected from the anus, followed by black liquid fæces. She experienced immediate relief, and all symptoms of irritation vanished.

Dr. Marcet analysed a biliary concretion which was here expelled, measuring better than an inch and a half in length, by an inch in diameter; 3.3 inches circumference. Mr. Thomas very rationally concludes, that this biliary calculus had been arrested in the sigmoid flexure of the colon, and there caused the obstruction in the bowels.

Art. 6.— *Case of Incontinence of Urine of Nine Years Duration, cured by external Pressure.* By John Hyslop, Esq.

The patient was a youth of 13 years of age, who for nine years, never passed a single day or night without several involuntary discharges of urine. Various means had been tried to remedy this evil, particularly tonics, cold bathing, blisters to the sacrum and perinæum, opiates, &c but without any success. Mr. Hyslop tried the jugum penis, but it did not answer the purpose. The following was his own contrivance.

"I selected a bougie of a size large enough to fill his urethra, from which I cut about two and a half, or three inches. Having placed that on the outside of the under part of the penis on a line parallel to the canal, with its point projecting a short way beyond the glans to avoid as much as possible any pain from pressure, I passed straps of adhesive plaster around, (first at the point of the penis, and afterwards continuing strap after strap the length of the piece of bougie,) and pulled them so tight as to press the bougie close in upon the urethra so that no space was left by which urine could pass. This was done at ten o'clock at night, and at three o'clock he called me out of bed, having a great desire to pass urine. I removed the straps, &c. and when he had emptied his bladder, I applied others in the same manner. The next desire for this evacuation was about seven o'clock, and the next again at eleven o'clock in the forenoon. After each evacuation the pressure was renewed without any unpleasant symptom and in three days he was cured of incontinence of urine."

How far such treatment might answer the purpose of cleanliness and convenience in paralysis, Mr. Hyslop leaves to the trial of future experience.

ART. 7.—*Case of Aneurism by Anastomosis in the left Orbit, cured by tying the common Trunk of the left carotid Artery.* By WILLIAM DALRYMPLE, Surgeon to the Norwich Hospital, &c.

This Aneurism commenced suddenly with intense pain in the left eye-ball, accompanied by a whizzing noise in the head. This occurred during pregnancy; and in labour, a tumor of an oblong form and bright red colour was projected between the eye-lids, occupying in a vertical direction, almost the whole space between the superciliary ridge and the lower edge of the ala nasi. This tumor was punctured several times by a surgeon, during the woman's confinement; it bled freely, and became smaller for a while, but afterwards increased and became so painful, as to render her life wretched. We shall not extract any part of Mr. Dalrymple's very minute description of this tumor during its subsequent growth, since it is impossible, without a drawing, to convey any distinct representation of it, and we much wonder Mr. Dalrymple omitted to give one.

"April the 7th, 1813. The operation was performed after the manner adopted by Mr. Astley Cooper, in the case of Humphrey Humphreys, (omitting, however, to pass the needle and thread through the artery, above one ligature and below the other) and its course was marked by the same circumstances which attended the operation of my distinguished friend. As soon as the margin of the mastoid muscle was raised, the descending nerve of the ninth pair was exposed to view, and when the sheath of the artery was laid open, the par vagum was seen at the outer side of the vessel. The jugular vein, pushed by the fore finger of the left hand beneath the edge of the mastoid muscle, afforded embarrassment. Nothing but the bare artery was included between the ligatures, which were formed of a small, but very strong *round* twine, and placed at a distance of about an inch and a quarter from each other. They were tied very firmly around the trunk of the artery, which was divided in the interspace, at the distance of about two thirds of an inch from the lower thread. The edges of the wound were brought together by the common adhesive strapping; and the end of one of the ligatures was marked for the sake of subsequent observation. The effects of the operation were immediate and decisive. As soon as the ligatures were tied, the pulsatory motions of the tumors on the forehead and cheek entirely ceased; but a slight thrilling was still perceptible in the tumid upper eye-lid. The red swelling of the lower eye-lid became paler and its surface shrivelled. A few minutes after the patient was placed in bed, she was quite free from pain, and the noise by which she had been so long tormented now also ceased; she declared to Mr. Stephenson, that "her head no longer felt like her old head."

By the 17th of May, the wound was nearly healed, and the patient was allowed to return to her family. By the 3d of July, the tumors had all disappeared, and the patient's general health seemed re-established. Two rather alarming hæmorrhages, however, took place subsequently, but they ceased spontaneously, and the patient finally recovered. This paper is very creditable to the professional character of Mr. Dalrymple.

ART. 8.—*Account of a Case, in which Parts of a Fœtus were found in a Tumor situated in the Abdomen of a Girl Two Years and a Half old.* By ED. PHILLIPS, M. D. of Andover.

This modest narrative occupies only three pages, and forms a pretty striking contrast to the costly volume of Mr. Highmore on a similar subject.

" A girl two years and a half old, was brought to me for advice in December last. Her mother informed me, that the child was born apparently healthy; but that in the third month she noticed an enlargement of the abdomen, which gradually increased without affecting the child's health or spirits. Two months previous to my seeing her, she had been attacked by frequent and distressing vomitings; she became emaciated; and the abdomen increased more rapidly in size. She lost her appetite and suffered much from pain: particularly when placed in the erect posture. Towards the conclusion of the disease, she was obliged to be always kept in the horizontal position. The mother had been informed that the child was dropsical, and aperient medicines had been ordered from time to time, without diminishing the size of the abdomen or allaying the pain and sickness. On examination, I found a hard regular tumor, situated on the left side of the abdodomen, which at first I thought to indicate a disease of the spleen: but on further inspection, I was led to alter this opinion, and became perfectly at a loss to explain the nature of the case. The superficial veins of the belly were much distended, as they generally are in dropsy; but no fluctuation could be perceived; the breathing was not disturbed; and the secretion of urine was undiminished. The child was obviously sinking under the disease; and I therefore gave the mother no hopes of her recovery, and simply prescribed some laxative powders, and the occasional use of clysters for the purpose of keeping open the bowels, which were in a torpid state. Three days afterwards I found all the symptoms greatly aggravated, and on the following day the child died. I prevailed on the parents to allow the body to be examined; and the dissection was made by my friend Mr. Pitman, nine hours after death. On opening the abdomen, a large tumor presented itself, occupying almost the whole of the left hypochondrium, and extending from the edge of the diaphragm nearly to the pelvis. The tumor was easily to be separated from the surrounding parts, with the exception of the left kidney, to which it was attached by a substance almost ligamentous. The liver bore marks of inflammation, and was studded with tubercles. The other abdominal viscera were healthy. The tumor, when removed from the body, might have weighed about eight or ten pounds. It was of an oblong shape, loosely covered by a delicate membrane highly vascular. On making a section of it, some ounces of a limpid fluid escaped from a cavity, the parietes of which were nearly cartilaginous; and in prosecuting the dissection, several similar compartments were discovered, all of which contained fluid or sanious matter. In the further examination of this fleshy mass, our attention was arrested by the resistance which the knife met with; and which led to the discovery of the bones which I have sent you. They were connected to the internal substance of the tumour by a structure decidedly muscular. The large bone resembling the tibia, was covered by muscle; the small bones, resembling those of the tarsus, were connected to the tibia by soft cartilaginous bands. From circumstances which it is unnecessary to explain, it happened that the dissection was conducted under peculiar difficulties, which prevented the examination of the body from being so minute as we wished it to be. But brief as the relation is, I conceived that it may be of some value to the physiologist, as it appears to furnish an additional instance of one fœtus being contained within another."

The bones referred to in this letter, are in Mr. Bell's Museum in Windmill street.

ART. 9.— *A case of Axillary Aneurism, for which the Artery was tied below the Clavicle.* By R. CHAMBERLAYN Esq.

"A muscular and healthy looking negro man, about 25 years of age, was sent to Kingston (Jamaica) on account of an aneurism in the left axilla, occasioned by a wound which he received from the point of a cutlas in the axillary artery, as he was carelessly carrying it, on or about the 15th of October, 18[illegible]4. The hæmorrhage from it was, as I was informed, profuse, and ceased in consequence of long continued syncope. The wound of the skin healed in three days without any recurrence of the bleeding. It was nearly nine weeks after the accident when he was first shewn to me. The cicatrix was scarcely perceptible. The tumor was the size of a large orange; and the pulsation in it very strong. The pain in the axilla from the pressure on the nerves was very distressing, but there was no œdema of the arm, nor any elevation of the clavicle. The pulsation in the radial artery was not so firm as in the other arm. He remained two weeks in town, during which time, I had frequent opportunities of seeing him, and the tumor appeared to me to be evidently increasing; was pushing out the latissimus dorsi, and showing itself behind. At this period he went into the country to see his relatives, and returned to town in ten or twelve days after. January 10th: The tumor had now considerably increased, and become much firmer, for when I first visited him, I could express the blood from the prominent part, which was in front of the axilla. The pulsation of the radial artery was becoming indistinct, and the pain in the axilla so severe as to deprive him of rest both night and day. The integuments over and about the aneurism were perfectly healthy in appearance. Upon the review of all the circumstances attendant on this case, it occurred to me that the operation was the only possible means that remained to rescue him from inevitable death, and that much danger was to be apprehended from a further postponement of it. Under these impressions, it was undertaken on Tuesday, January 17, 1815, in the presence of Mr. Garcia and Mr. M'Bravo, surgeons in this city. The patient was placed upon an operating table, with a pillow under his shoulders, and his head supported A transverse incision of three inches in length was made through the skin and platysma myoides along and upon the lower edge of the clavicle, three fingers' breadth from the external extremity of that bone, and terminating about an inch from the acromium scapulæ. This incision divided a small artery, which was immediately secured. A second incision of three inches in length was also made obliquely through the integuments over the deltoid and pectoral muscles, meeting the first nearly in the centre. The cellular membrane and fat lying between them at the upper part were now removed. The next step in the operation consisted in detaching the clavicular portion of the pectoralis major, and taking away the fat and cellular membrane lying over the subclavian vessels. The artery was now brought into view, and its pulsation made it clearly distinguishable from the contiguous parts; but I could not detach it, nor pass the ligature under it with the facility I expected, from its depth. After several ineffectual efforts, I succeeded in conveying the ligature under it, by means of an eyed ball probe previously curved for the purpose, and bringing up its point with a pair of forceps, tied the artery as it emerges from under the clavicle to proceed to the axilla. The drawing the knot was attended with little pain; the wound was closed by the dry suture, and the patient returned to his bed."

On the 30th of January, the ligature came away spontaneously, and the granulations being prominent, were touched with the sulph. cupri. The temperature of the two arms was equal—the patient got out of his bed and walked about. On the 22d February, the wound closed, and the aneurismal tumor was about as large as a turkey's egg, very solid, but void of pain.

This operation is certainly very creditable to Mr. Chamberlayn.

**Art. 10. — *Successful Treatment of a Case of Cynanche Laryngea.* By James Watson Roberts, M. D. of Bishop-Stortford.**

The subject of this case was an army physician, who, having spent the evening of the 16th of July, 1794, in camp, where he drank freely, was called up in the night to visit a general officer, and caught cold. Three days afterwards, Dr. Roberts was summoned to him, and found the face much swollen, and redder than usual; the eyes protruding and blood-shot; fulness of the neck; breast suffused with a purplish colour. There was no redness of the fauces, enlargement of the tonsils, or difficulty of deglutition. He referred all his complaints to the pomum Adami. The skin was very dry, the temperature increased; the pulse 112, full, and laborious; the carotids beating forcibly; voice hoarse; no cough. The dyspnœa was not constant, nor was there any symptom indicative of inflammation in the lungs. He had great somnolency; falling asleep even while spoken to. As soon as his eyes were closed, he began to snore, when his face was instantly covered with a crimson suffusion, and he awoke, seemingly in a fright, gasping for breath, and casting both arms out of bed, with the palms extended towards the byestanders, looking most anxiously for assistance, being unable to utter a word.

In this painful struggle, the inhalation of oxygen gas gave instantaneous, but only temporary relief; the paroxysms recurred as often as the patient fell asleep; the disposition to which was invincible. Sixteen ounces of blood were taken from the arm, by which he was considerably relieved. He took a cathartic of salts and senna. The relief was but temporary. At mid-day, the pulse was full, hard, and strong, and the sense of suffocation as distressing as ever.

A larger venæsection was employed with manifest advantage, for he could afterwards sleep for a few minutes together without the sense of suffocation supervening. A large blister was applied to the breast. In the evening a pleasing change had taken place: the sense of suffocation was neither so frequent nor so urgent; the vascular action was reduced, and the skin was cooler. The cathartic had operated freely, and the blister had risen; but the most flattering circumstance was, that the patient had enjoyed some refreshing and undisturbed repose. Draughts with acetate of ammonia and tartrite of antimony were ordered for the night. He experienced frequent attacks before morning. 20th. Complains now of head-ache, is feverish, pulse full and quick. The blood last drawn is highly inflamed; urine high coloured. Another large venæsection.

"From this period safety seemed to be ensured, as he could shortly after lie upon either side, and sleep undisturbed for an hour together, without being seized with the spasmodic affection of the throat, where he merely felt a dull pain, and uneasy turgesence, which gradually subsided under the use of purgatives and the antiphlogistic regimen." 139

The same gentleman was attacked with this formidable disease fourteen years afterwards, but being differently treated it proved fatal.*

Some very sensible and highly interesting remarks are made on this case by the learned president of the society. Of eight cases that have been lately recorded of this disease, six proved fatal. The president suggests the propriety, in laryngeal inflammation, of applying *very large* blisters to the thorax; of fomentations or poultices to the throat itself; of copious and early venæsection; of total abstinence from opiates.

We may here add, that we ourselves have witnessed two cases of this disease, one of which proved fatal, and the other was cured. The first case was that of an officer who swallowed some ice cream while in a violent heat under a tropical sun. This was followed by laryngeal inflammation of which he died about the 11th or 12th day. We did not see him till within three days of his death, when all remedies were in vain. He had not been bled; but blisters, purgatives, &c. had been used. We opened the body, and found the membrane investing the larynx inflamed, and in many parts suppurated. A considerable quantity of this purulent matter had descended into the bronchial tubes, from whence it could not be expectorated, in consequence of the state of the larynx. From circumstances unnecessary to relate, this fatal case made a deep impression on our minds; and not many months afterwards, a case of a similar nature presented. We bled twice *ad deliquium*; applied a *large blister* to the throat and upper part of the chest; kept up a hypercatharsis with calomel, extract of colocynth, and tartrite of antimony: immersed, several times in the day, the lower half of the body in the warm bath, and applied mercurial

* Vide 3d Vol. of Transactions of a Society, &c. Art. 22.

frictions to the thighs. In three days the complaint was completely subdued. This happened about twelve years ago, and we candidly confess, that we acted on the supposition of the disease being a species of croup, and without making the nice distinction between tracheal and laryngeal inflammation. We hope, however, that these cases, imperfect as they are, will corroborate the propriety of the evacuating system.

Art. 11.—*Account of a Case of Croup, in which the Operation of Bronchotomy (Tracheotomy) was successfully performed.* By Thos. Chevalier, Esq. &c.

As this is a short and interesting paper, we shall extract it entire, as a conclusion to the first part of our analysis.

"Master B., aged 7 years, had been much disordered in his stomach and bowels, and had a slight degree of fever. This, however, had so far subsided, that his parents intended to return to their home in the country. Mr. Lightfoot, who had attended him, called in the morning of April 25, 1814, and was told that the child had a little cold and cough; he recommended that if the complaint should increase, leeches should be applied to the chest, and then a blister. In the evening his assistant informed him, that leeches and a blister had been sent for. Mr. L. was desired to call again on the following morning, when he found the breathing distinctly manifesting that croup had taken place. He instantly bled him largely from the jugular vein, and as soon as he recovered from the fainting which this produced, directed an emetic, which operated freely, and occasioned him to bring up a portion of a membranous substance, which appeared evidently to Mr. L and Dr. Merriman, who was now called in, to resemble that found in the trachea of those who die of this disease. A purgative was given, and calomel was ordered to be taken every six hours. On the afternoon of Wednesday, the 27th, the difficulty of breathing greatly increased, the countenance grew livid, cold sweats came on, and he appeared sinking. He was nevertheless perfectly sensible. No chance of his recovery now seemed to remain, unless it were by opening the trachea, and I was applied to for this purpose. I exposed the trachea just below the cricoid cartilage, and divided two of the cartilaginous rings vertically, cutting afterwards transversely in the interstice between them. About an ounce, or an ounce and a half of a reddish, brown, and frothy mucus, gushed out through the opening, and by a tolerably full inspiration which presently followed, the child was enabled to cough up more of the same kind. The breathing became immediately relieved, the cold sweat ceased, and the countenance in a short time resumed much of its natural appearance. The pulse was 160. On the morning of the 28th, the breathing was much improved: pulse 144. He was directed to take half a drachm of oxymel of squill every hour in a little camphor mixture; and four ounces of the camphor mixture were to be employed as an enema, several times in the course of the day. In the evening his breathing had become quite easy, and the cough much less sonorous. On the 29th, he brought up with a slight cough about a desert spoonfull of tough mucus, but it did not appear that any had passed through the wound. He was better in all respects, and from this time his recovery was gradual and uniform, so that on the 13th May, he was sufficiently well to return to his home in the country. Since this case occurred, I have had an opportunity of witnessing that of a child, 5 years old, in which the same operation was performed by my friend Mr. Blair. In this instance, the first symptoms of disease were fever with an erysipelatous state of the tonsils, from whence inflammation extended into the trachea, producing impeded respiration. Suffocation was rapidly advancing, and the operation was deemed the only resource. The trachea was opened longitudinally, and more than an ounce of red and frothy mucus issued through the wound; this was followed by a pretty full inspiration, and a subsequent cough, in which much more of the same sort of mucus was brought up. The bronchia appeared then to be entirely cleared; for the breathing became free and easy, and the child was for a short time greatly relieved; but the debility which had been previously induced by the disease was too great to be surmounted, and occasioned death the same afternoon.

In such cases of croup as I have examined after death, I have found the trachea choaked up with this mucus, and I am very much inclined to suspect that it is by it, more than by the coagulable lymph, that suffocation is finally produced. When the quantity of mucus is such as to prevent the air from getting into the branches of the bronchia, and from them to the air cells, the patient becomes unable to clear the tube by coughing, the properties of the blood must rapidly degenerate, and death will speedily ensue. But I doubt whether this is so often to be ascribed to such an accumulation of coagulable lymph itself, as absolutely precludes the transmission of air. If this suspicion be just, the use of bronchotomy must result chiefly from its emptying the trachea of mucus, and thus enabling the patient so to cough, as to clear the branches of the bronchia, without which the air, even if it pass through the obstructed larynx, cannot reach the lungs. It would also follow, that the introduction of a canula, or tube, into the trachea after the operation, for the sake of securing the passage of air, is of less consequence than has been usually supposed, and might even be better omitted, as the presence of an extraneous body must irritate the internal membrane, and would thus be likely to increase that secretion of mucus, from an accumulation of which the principal danger is to be apprehended."

(To be continued.)

